# Parameters of Somatosensory Evoked Potentials in Patients with Primary Sjӧgren's Syndrome: Preliminary Results

**DOI:** 10.1155/2018/8174340

**Published:** 2018-04-05

**Authors:** Edyta Dziadkowiak, Agata Sebastian, Małgorzata Wieczorek, Elżbieta Kusińska, Marta Waliszewska-Prosół, Piotr Wiland, Maria Ejma

**Affiliations:** ^1^Department of Neurology, Wroclaw Medical Hospital, Borowska 213, 50-556 Wroclaw, Poland; ^2^Department of Rheumatology and Internal Medicine, Wroclaw Medical University, Borowska 213, 50-556 Wroclaw, Poland; ^3^Department of Geoinformatics and Cartography, Institute of Geography and Regional Development, University of Wroclaw, Uniwersytecki 1, 50-136 Wroclaw, Poland; ^4^Department of Neurology, Wroclaw Medical University, Borowska 213, 50-556 Wroclaw, Poland

## Abstract

Primary Sjogren's syndrome (pSS) is a chronic autoimmune disease. The aim of the study was to establish whether in patients with pSS without central nervous system (CNS) involvement, the function of the central portion of the sensory pathway can be challenged. In 33 patients with pSS without clinical features of CNS damage and normal head computed tomography scan, somatosensory evoked potentials (SEP) were studied. The results were compared to other clinical parameters of the disease, particularly to immunological status. The control group consisted of 20 healthy volunteers. Mean latency of all components of SEP was considerably prolonged in patients compared to the control group. Mean interpeak latency N20-N13 (duration of central conduction TT) did not differ significantly between the groups. However, in the study group, mean amplitude of N20P22 and N13P16 was significantly higher compared to healthy individuals. In patients with pSS, significant differences in SEP parameters depending on the duration of the disease and presence of SSA and SSB antibodies were noted. The authors confirmed CNS involvement often observed in patients with pSS. They also showed dysfunction of the central sensory neuron as a difference in the amplitude of cortical response, which indicates subclinical damage to the CNS.

## 1. Introduction

Primary Sjogern's syndrome (pSS) is a chronic autoimmune exocrinopathy characterized by dysfunction of exocrine glands resulting from lymphocytic infiltration. Inflammation can occur in other organs as well. The pathogenesis of this disease is not fully understood. pSS affects 0.09–3.5% of the general population, and morbidity is estimated at 3.9–5.3/100000 population. Females are more often affected [1, 2]. Concomitance of pSS and B-cell lymphomas is also observed [3].

Central nervous system (CNS) involvement is estimated to be present in 2.5–60% of patients with pSS. The injury of the peripheral (10–60%) part is more common than that of the CNS (20%) [1–3]. Very rarely (4%), both peripheral and CNS are affected in the same patient [4]. Sensory neuropathy (50–60%), particularly sensory neuropathy of thin fibers, motor sensory neuropathy, and sensory ataxia are the major types of dysfunction. Less commonly, multifocal mononeuropathy (6–12%), polyradiculoneuropathy (4–14%), cranial neuropathy (17%), and autonomic neuropathy (50%) can develop [5, 6].

CNS involvement could have many clinical presentations and various severities. It can be presented as encephalitis, neuromyelitis optica spectrum disorder (NMOSD), or mild episodes of headache or emotional disorder [7, 8]. Radiological images, for example, computed tomography (CT) and magnetic resonance (MR), are necessary for the assessment of severe CNS involvement. In subclinical benign cases, functional testing, for example, evoked potential of variety modality, is helpful.

Somatosensory evoked potential (SEP) study is one of the most sensitive and objective neurophysiological methods used to evaluate the function of afferent sensory pathways. SEPs are generated at different levels of the sensory pathway leading to the primary sensory cortex through the dorsal column and medial lemniscus. N9 notch is generated by the brachial plexus, and N13 by postganglionic fibers of the first sensory neuron, that is, posterior radix and/or dorsal column of the cervical spinal cord, while N20 originates in the primary sensory cortex of the contralateral parietal lobe, at the location of somatotopic representation of the hand.

The aim of the study was to establish whether in patients with pSS without symptoms of central nervous system involvement, the dysfunction of the central portion of the neurosensory pathway can be observed. The researchers also analyzed whether rheumatological prognostic factors (including skin lesions indicating vasculitis and laboratory parameters reflecting disease activity) correlate with SEP parameters.

### 1.1. Subjects

The study group consisted of 33 patients (including 1 male) with a mean age of 50, who fulfilled the diagnostic criteria of pSS at the time of SEP study, according to the American-European classification (2002) [9]. Retrospectively, the patients met the diagnostic criteria of pSS since 2016 [10], which were unavailable at the time of the study. The exclusion criteria included a history of neurological, metabolic, and deficiency disorders and use of drugs affecting central nervous system activity. Concomitant diseases included lipid disorder (1 patient), cholelithiasis (1 patient), controlled hypertension (3 patients), and euthyroid struma (9 patients).

The control group consisted of 20 healthy volunteers selected with respect to sex and age.

## 2. Methods

In all patients, neurological history was taken and physical examination was performed, as well as head CT scan. Fatigue was assessed using the FACIT questionnaire and visual analogue scale (VAS; 0–10 pts, where 0 denoted the total lack of symptom, while 10 referred to intensity affecting everyday home activity). The analysis of pSS activity was conducted using EULAR Sjogren's Syndrome Disease Activity Index (ESSDAI), EULAR Sjogren's Syndrome Patient Reported Index (ESSPRI) scales as well as focus score in minor salivary glands biopsied from the lower lip [11], and laboratory parameters including C3 and C4 component levels, antinuclear antibodies (ANA; measured by indirect immunofluorescence, IF), rheumatoid factor (RF), erythrocyte sedimentation rate (ESR), C-reactive protein (CRP), peripheral blood morphology, and total protein level in serum.

The evoked potential study was conducted using the Viking Quest equipment. The procedure was conducted according to the International Federation of Clinical Neurophysiology (IFCN) guidelines [11, 12]. The study was conducted in the supine position, in a quiet and dimmed room at 22–24 degrees Celsius. Superficial Ag/AgCl electrodes with a diameter of 10 mm by Nicolet Instrument Corporation were used and placed on the skin of the head according to the international 10–20 scheme and fixed using the adhesive-conductive Ten20 Conductive paste by D.O. Weaver and Co.

SEPs were achieved by stimulating median nerves with transdermal electric impulses. Three superficial sensory electrodes were placed at (1) Erb's point (mid-clavicular point) with contralateral reference electrode; (2) at the level of C7 vertebra with reference electrode at Fz central point; and (3) on the head skin above the cortex representation of the hand, in the right (C4/P4) and left (C3/P3) parietal regions, on the contralateral side to the stimulated median nerve with reference electrode in the frontal region at Fz point. Ground electrode was placed above the stimulating electrode on the forearm. The stimulating electrode on the wrist generated impulses with a duration of 100 ms and a frequency of 4.7 Hz and intensity resulting in thumb movement in the range of 1-2 cm. Three responses were averaged, and the analysis time was 100 ms. Each registration was performed twice in order to confirm repetitiveness of the measurement.

Responses were selected, and their characteristic components were interpreted. Latency of SEP components were analyzed: peripheral—N9 and N10, brainstem—N13 and P16, cortical—N20 and P22, and interpeak latency—N20-N13, that is, central conduction time TT. Also, the amplitudes of N9/P10, N13/P16, and N20/P22 were assessed.

The study was conducted in accordance with the principles of the Declaration of Helsinki. The locally appointed ethics committee has approved the research protocol (357/2010), and informed consent has been obtained from all the subjects.

### 2.1. Statistical Analysis

Statistical analysis was conducted using STATISTICA 12.0 software. All tests (for normality, homogeneity of variance, equivalence of means, and ranked tests) were conducted at the significance level of *α* = 0.05. To test the normality of distribution, the Shapiro-Wilko test was used. Variables with normal distribution were tested for homogeneity of variance. After positive verification of both hypotheses (of normal distribution and homogenous variance), the hypothesis of the equivalent means between both groups was tested using Student's *t*-test. The comparison of variables, the distribution of which was not normal according to the Shapiro-Wilk test, was conducted using the ranked Mann–Whitney *U* test.

For all multiple testing, Bonferroni's correction was used [13]. Ten variables are tested, therefore the particular significance level of *α* = 0.005.

## 3. Results

### 3.1. Analysis of Rheumatological Parameters

In the study group, the mean time since the diagnosis of pSS to the SEP study was 4 years (1–14 years). The first symptoms of pSS included xerophthalmia and/or xerostomia (63%), fatigue (24%), arthralgia lasting over 30 minutes per day (21%), skin lesions typical for pSS [14] (12%), peripheral arthritis of the extremities (9%), and large swollen salivary glands (6%). At the time of the study, symptoms of arthritis were present on 28 (85%) patients, respiratory involvement in 19 (57%), swelling of major salivary glands (parotid/submandibular glands) in 14 (42%), skin lesions typical for pSS in 10 (30%), and lymphadenopathy in 5 (15%).

The extent of inflammatory infiltration expressed as the mean focus score was 2.1 (0–5) in patients while the ESSDAI score was 20 points (4–24 pts), which indicated the high activity of the disease [15]. In all patients, mild xerophthalmia according to ESSPRI scale 5.2 (2–8) persisted in all patients, while mild xerostomia rated 5.2 (0–9) on ESSPRI scale was noted in 31 patients (94%).

ANA antibodies ≥ 1 : 320 were present in 26 (78%) patients with pSS, anti-SSA/Ro60 in 26 (78%), anti-Ro52 in 22 (66%), and anti-SSB/La in 21 (64%). In 3 cases (9%), the presence of RF was not confirmed.

No correlation between pSS activity expressed on the ESSDAI scale and the duration of the disease was found (*p* = 0.18).

All laboratory results are summarized in Table 1. None of the pSS patients showed anemia or thrombocytopenia.

32 patients (94%) were treated with chloroquine (250 mg per day) or hydroxychloroquine (200 mg or 400 mg per day), 28 (82%) with corticosteroids, 6 (18%) with azathioprine, 4 (13%) with cyclosporine, and 5 (15%) with methotrexate.

### 3.2. Neurological Examination

At the time of the study, all patients reported fatigue lasting for over 3 months, 7 patients complained about sleep disturbances, 7 had low mood, anxiety, and fear, 4 suffered headaches, and one had a history of optic neuritis (which was the primary manifestation of pSS). Mean fatigue assessed using the visual analogue scale (VAS) was 6.7 points (4–10 pts).

On neurological examination, primitive reflexes (snout, palmomental reflex grasping) were noted in 6 patients, and slightly weakened ankle jerk reflexes were observed in 12. Otherwise, the neurological status of the patients was normal, and especially, no dysfunction of position sense, vibration, stereognosis, and two-point discrimination was found. In the patients, no abnormalities on the head CT scan were found. All patients had chronic fatigue syndrome, and 20 patients had normal neurological status (Table 2).

### 3.3. SEP

**Figure 1:**
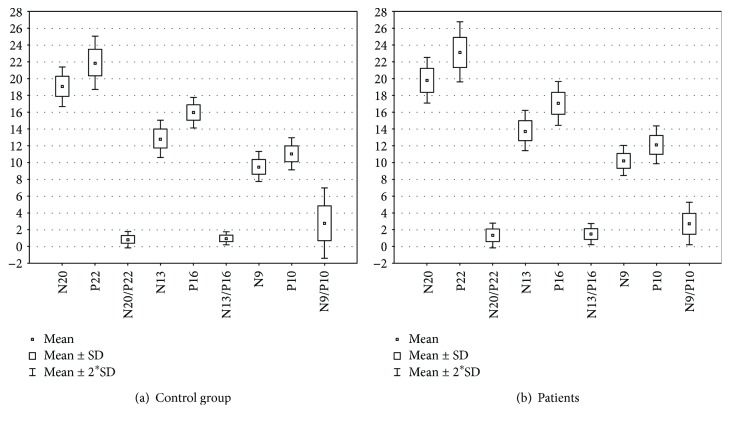
Box plot diagrams for SEP parameters in the (a) control group and (b) study group. Mean: mean standard deviation.

SEPs were obtained in all patients and all healthy individuals in the control group. The mean latency of all SEP components was considerably longer in all patients compared to the control group (Table 3). Interpeak N20-N13 (central conduction time TT) did not differ significantly between both groups. In the study group, mean N13/P16 and N20/P22 amplitude was significantly higher compared to healthy individuals; however, no statistically significant difference was found with respect to N9/N10 amplitude (Table 3, Figure 1).

In pSS patients, significant differences in SEP parameters were observed depending on the duration of the disease and the presence of anti-SSA/SSB antibodies. Patients suffering from the disease for more than 10 years compared to patients with a shorter duration of the disease showed higher N9/P10 amplitude (1.2 versus 1.9; *p* = 0.0026). Patients with anti-SSA antibodies (1.4 versus 2.3; *p* = 0.0016) and anti-SSB antibodies (1.3 versus 2.0; *p* = 0.0034) showed significantly lower N13/P16 amplitude.

No statistically significant differences in mean SEP parameters were found depending on the presence of skin lesions, xerophthalmia, joint pain and swelling, focus score, C3 and C4 component levels, ESR, CRP, presence of Ro52 antibodies, and treatment. Patients with joint pain lasting more than 10 years show longer P10 latency (13.2 versus 11.9; *p* = 0.008).

## 4. Discussion

In patients with CNS involvement, in 50% of the cases, the first manifestation of pSS is neurological deficit; however, Delalande et al. [16] in a study on 82 patients showed that neurological symptoms preceded the diagnosis of pSS in 81% of cases. Karaca et al. [17] presented 11 patients, in whom the first symptoms were neurological ones: in 7 patients, CNS involvement manifested as MS-like form and optic neuritis, and in 4 cases, peripheral nerve injury as Guillain-Barre syndrome and multifocal mononeuropathy. In 2 patients, increased SEP latency was observed and in 1 reduced SEP amplitude.

In the literature from the past 20 years, there are only few reports presenting SEP in patients with pSS [18–20]. They cover single cases or very limited groups of patients. For instance, Bouraoui et al. [21] presented a case of a 67-year-old female patient with pSS, in whom reduced pinprick and vibration sensation were observed in lower extremities. In that case, SEP of tibial nerves showed prolonged latency of P40 cortical wave. However, the results of SEP study in pSS are inconsistent. Some publications describe prolonged SEP latency [19, 22], while others present normal SEP [20]. Our study covers a larger population of patients and analyzes SEP parameters in 33 patients. In all patients, the head CT scan was normal, 6 patients presented with frontal release signs, and 12 presented with weakened ankle jerk reflexes without other signs of neuropathy or CNS damage. Only using SEP, which is a sensitive neurophysiological method, allowed to detect bioelectric disturbances in both peripheral and central portions of the sensory pathway. In the study group, prolonged SEP latency at each level—brachial plexus, brainstem, and cortex—was observed. Prolonged latency of response can result from peripheral injury since there were no significant differences in central conduction time TT between the study and the control group. The lack of such differences indicates normal conduction of sensory impulses within the contralateral medial lemniscus and the hypothalamic-cortical tract. However, significantly higher amplitude of cortical response (N20/P22) demonstrates dysfunction of central neurons.

Increased amplitude of cortical response suggests increased excitability of the cortex and lowered seizure threshold. This phenomenon can be observed in autoimmune disorders, where the risk of developing epilepsy is even five times greater [23]. In pSS, seizures can be one of the neurological manifestations of the disease [24–26]. Attout et al. [24] described a 70-year-old female patient with paroxysmal loss of consciousness without clonic movements, in whom paroxysmal focal seizures were observed on electroencephalogram (EEG) from the left anterior electrodes extending to the contralateral side during the HV test.

Introduction of valproic acid resulted in regained consciousness. Seizures in pSS are often drug-resistant and do not show characteristic morphology or primary location. Their pathogenesis can be related to various etiological factors, inflammation, vascular lesions, and antibody production [23]. Although none of our patients presented with epilepsy, the mean amplitude of somatosensory cortical response was significantly higher compared to the control group. Such observations have not been described for pSS so far. However, in patients with myoclonic epilepsy, high amplitude of N20/P25 complex, so-called gigantic SEP, was observed [27, 28]. The pathomechanism of this phenomenon is unclear. It can suggest disturbed inhibition occurring directly after excitation. There is a hypothesis of possible “wandering” impulse between the primary motor cortex and the primary sensory cortex.

Higher N13/P16 and N20/P22 amplitudes in patients with pSS compared to the control group proved in our study may be associated with glutamatergic excitatory amino acids (EAA). Many publications in recent years indicate the role of EAA receptors in the conduction of sensory and pain impulses, located in spinal nerve ganglions and sensory pathways [29]. It was established that the system contributes to the development of inflammation and rheumatic diseases [30]. The significant role of EAAs in inflammation and pain conduction at the level of the spinal cord and brain was proved in experiments conducted on animal models. For example, two days after the administration of an inflammation-triggering factor, an increase in the number of different glutamatergic receptor subtypes (AMPA, NMDA, KA) in peripheral myelinated and nonmyelinated nerve endings was proved [31]. The relationship between chronic pain and cognitive disorders has been established [32]. Although the influence of pain on neurological complications in pSS is not well known these days, it has been proven that in pSS cognitive dysfunction is the characteristic for subcortical lesions of the frontal lobe [33]. As far as we know, our research is the first one indicating an association between pain and SEP outcomes. Comparing pSS patients with varying durations of arthralgia, we found longer latency of P10 peripheral response at Erb's point. This indicates impaired conduction of the sensory stimulus along the peripheral neuron. It is possible that some cytokines or even nociceptors contributing to pain response caused by inflammation also indirectly play part in the damage of the nervous system. Hence, early treatment of the active forms of the pSS with the involvement of the musculoskeletal system is important in order to stop inflammation. This hypothesis, however, requires further observations and well statistical analysis due to the small number of the study group.

The literature describes the relationship between different immunological profiles among patients with pSS and the development of neurological complications, especially nonataxic sensory neuropathy [5, 29]. Predisposing factors include the presence of ANA antibodies, including anti-SSA/SSB antibodies and RF, as well as hypergammaglobulinemia [5]. Our study also proved, as previously reported, the relationship between the presence of anti-SSA/SSB antibodies and SEP parameters. Significantly lower N13/P16 amplitude was reported in patients with the anti-SSA/SSB antibodies compared to patients without such antibodies. It points out the significant role between inflammation and damage to sensory neurons in pSS. Interestingly, SEP changes did not correlate with lowered values of the C3 and C4 complement components and the intensity of minor salivary gland infiltration expressed by the focus score, which may be an evidence of high disease activity. ESSDAI was initially higher in patients with pSS. Although SEP examination is not part of ESSDAI evaluation, our results show that this examination should consider patients with pSS for the identification of microtraumas of the nervous system. Also, the relation between the presences of purpura, which may be a sign with vasculitis, was not proven, while the damage of small vessels is considered by some authors to be crucial to neuronal damage in pSS [4]. Our study is limited by a lack of cryoglobulinemia testing (due to technical reasons), which is an independent factor of sensorimotor neuropathy and mononeuritis multiplex.

Some authors proposed the role of muscarinic receptor activation in the pathogenesis of pSS and the modulation of certain central nervous system function, for example, cognitive functions [34, 35]. The precise immune mechanisms remain still unclear. Reina et al. postulate that early agonistic-promoting activation in two types of muscarinic acetylcholine receptors (mAChRs) M1 and M3 initiated by antibodies binds to persistently activate cerebral frontal cholinoceptors. This activity might induce desensitization, internalization, and/or intracellular degradation of the mAChR, leading to a progressive decrease of cerebral M1 and M3 mAChR expression and activity. They hypothesized that the CUN manifestations in SS might be induced by an impaired response to cholinergic stimuli by mAChR antibody-specific interactions [36].

Our results showed some SEP differences depending on the duration of pSS. Significantly higher amplitude of N13/P16 was reported in patients with pSS lasting more than 10 years compared with a shorter history of pSS, despite the fact that we did not observe any correlation between ESSDAI. This may be an evidence of insufficient inhibition of pathological processes despite therapy. The influence of the disease duration on evoked potentials has been proved earlier by analyzing P300 endogenous potentials in pSS [37]. It was found then that latency of N200 and P300 components is significantly extended in patients with pSS lasting longer than 10 years, which indicates greater cognitive deficit.

Our results showing prolonged mean latency of all SEP components in patients compared to the control group proved the involvement of the peripheral nervous system, which is often observed in pSS. Despite no differences in central conduction time, significant changes of cortical response amplitude showed dysfunctions of the central sensory neuron, which corresponds to subclinical damage of the CNS. We observed the relationship between SEP parameters and duration of pSS, duration of arthralgia, and presence of anti-SSA and SSB antibodies. Those results, however, require further observations in a larger number of pSS patients.

## Figures and Tables

**Table 1:** Selected laboratory parameters in pSS patients.

	Number of patients (*n*/%)	Normal values
Decreased level of C3 component	6/18%	0.9–1.8 g/l
Decreased level of C4 component	4/12%	0.1–0.4 g/l
Hypergammaglobulinemia^∗^	10/30%	0.7–1.4 g/dl
Elevated ESR, mm/h	10/30%	1–20 mm/h
Elevated CRP	3/9%	0–5 mg/l
Lymphopenia	19/57%	1.5–3.5 k/ul

^∗^Hypergammaglobulinemia was measured by electrophoresis of serum.

**Table 2:** Neurological assessment.

	Number of patients (*n*/%)
(1) Subjective syndrome:	
(i) Sleep disorder	7/21%
(ii) Depressed mood	7/21%
(iii) Headache	4/12%
(iv) Optic neuritis	1/3%
(2) Changes in neurolocical examination:	
(i) Primitive reflexes	6/18%
(ii) Adynamic ankle jerk	12/33%

**Table 3:** SEP latency and amplitude in pSS patients and in the control group. These are significant where *p* value < 0.005 (Bonferroni adjustment).

SEP	Study group *n* = 33	Control group *n* = 20	*p* value
Value	Mean ± SD	Mean ± SD	
*Latency (ms)*			
N9	10.26 ± 0.90	9.51 ± 0.91	*0.009*
P10	12.23 ± 1.13	11.05 ± 0.96	*0.0002*
N13	13.74 ± 1.20	12.80 ± 1.10	*0.0054*
P16	16.97 ± 1.30	15.95 ± 0.89	*0.0014*
N20	20.07 ± 1.37	19.06 ± 1.17	*0.0136*
P22	23.18 ± 1.77	21.90 ± 1.60	*0.0050*
TT (N20–N13)	6.29 ± 1.22	6.26 ± 0.85	0.8507
*Amplitude (μV)*			
N9/P10	2.76 ± 1.26	2.79 ± 2.10	0.673
N13/P16	1.53 ± 0.62	0.99 ± 0.44	*0.0005*
N20/P22	1.29 ± 0.74	0.81 ± 0.47	*0.0030*
